# Numerical bifurcation analysis of turing and symmetry broken patterns of a PDE model for vegetation dynamics

**DOI:** 10.1007/s00285-026-02384-4

**Published:** 2026-04-09

**Authors:** Konstantinos Spiliotis, Lucia Russo, Constantinos Siettos, Francesco Giannino

**Affiliations:** 1 Laboratory of Mathematics and Informatics (ISCE), Department of Civil Engineering, Democritus University of Thrace, Komotini, Greece; 2 Istituto di Scienze e Tecnologie per l’Energia e la Mobilitá Sostenibili, CNR, Naples, Italy; 3 Dipartimento di Matematica e Applicazioni “Renato Caccioppoli”, Universitá degli Studi di Napoli Federico II, Naples, Italy; 4 Dipartimento di Agraria, Universitá degli Studi di Napoli Federico II, Naples, Italy

**Keywords:** Numerical Bifurcation Analysis, Symmetry Breaking in PDEs, Turing Instabilities, Reaction-Diffusion Ecological Systems, 65P30, 35K57, 35B36, 37M20, 37M20

## Abstract

We study the mechanisms of pattern formation for vegetation dynamics in water-limited regions. Our analysis is based on a set of two partial differential equations (PDEs) of reaction–diffusion type for the biomass and water, and one ordinary differential equation (ODE) describing the dependence of the toxicity on the biomass. We perform a linear stability analysis in the one-dimensional finite space, we derive analytically the conditions for the appearance of Turing instability that gives rise to spatio-temporal patterns emanating from the homogeneous solution, and provide its dependence with respect to the size of the domain. Furthermore, we perform a numerical bifurcation analysis in order to study the pattern formation of the inhomogeneous solution, with respect to the precipitation rate, thus analyzing the stability and symmetry properties of the emanating patterns. Based on the numerical bifurcation analysis, we have found new patterns, which form due to the onset of secondary bifurcations from the primary Turing instability, thus giving rise to a multistability of asymmetric solutions.

## Introduction

It is well known that the self-organized spatio-temporal patterning of vegetation, especially in water-limited regions, comes as a feedback response to ecosystem stability and species diversity (Vincenot et al. 2016; Zhao et al. 2019; Inderjit et al. 2021). Thus, the demystification of the mechanisms that govern the formation and dynamics of such spatiotemporal vegetation patterns is at the forefront of contemporary ecological and environmental research efforts (Kéfi et al. 2007; Tarnita et al. 2017). An important open research question revolves around the relation between vegetation patterning changes/disturbances and catastrophic/irreversible transitions, both in the environmental landscape and biodiversity. For example, Kéfi et al. (2007) showed that patch-size distributions in arid Mediterranean ecosystems may serve as early-warning signals for the onset of desertification. Bonanomi et al. (2014) suggested that vegetation rings facilitate the diversity of species. Zhao et al. (2019) showed that the patchy vegetation in salt marsh ecosystems promotes species biodiversity. Such patterns include but are not limited to stripes, spots, rings, labyrinth-like structures and spiral waves (Inderjit et al. 2021).

To explain such self-organizing spatio-temporal patterns, various mathematical dynamical models have been used ranging from microscopic models, including stochastic cellular automata (Silvertown et al. 1992; Pascual et al. 1421; Kéfi et al. 2007; Russo et al. 2014), and agent-based/individualistic models of deterministic ordinary differential equations (ODEs) (Vincenot et al. 2016, 2017), to continuum models of partial differential equations (PDEs) (Bonanomi et al. 2005; Cartení et al. 2012; Marasco et al. 2014; Severino et al. 2017). The keystone idea that underpins the above mathematical models is that of the “scale-dependent feedback” (Rietkerk and Koppel 2008) mechanism between species and limited resources. This mechanism is governed by the so-called activator–inhibitor principle introduced by Turing in his celebrated 1952 paper “The chemical basis of morphogenesis" (Turing 1952, 1990) on the spontaneous formation of patterns in diffusion-reaction systems (see also the discussion in Maini et al. (1997); Rietkerk and Koppel (2008); Ball (2015); Krause et al. (2021)).

What is usually done with such continuum-level vegetation reaction-diffusion PDEs, is temporal simulation and linear stability analysis (see e.g. Klausmeier (1999); Cartení et al. (2012); Marasco et al. (2014); Gowda et al. (2016)) of the homogeneous (spatial independent) dynamics (Marasco et al. 2014; Gowda et al. 2016). However, simple temporal simulations and/or linear stability analysis are not adequate for the investigation of far-from-the-equilibrium nonlinear phenomena. For example, in several studies it has been shown that Turing instabilities may experience secondary bifurcations leading to far-from-equilibrium oscillating solutions (Lamb et al. 2006; Spiliotis et al. 2018), spatio-temporal chaos (Aragón et al. 2012; Banerjee and Banerjee 2012) and symmetry-breaking bifurcations (Barrio et al. 2002). In such regimes, nonlinearities play a key role not only in stabilizing a pattern, but also in producing unsuspected bifurcations lined with catastrophic transitions (Russo et al. 2019; Spiliotis et al. 2018, 2021). Thus, to systematically investigate such phenomena systematically, the exploitation of the full arsenal of numerical bifurcation theory is of utmost importance (Satnoianu et al. 2000; Henderson et al. 2016; Russo et al. 2019; Spiliotis et al. 2021)).

Here, we construct the full bifurcation diagram of a vegetation model consisting of two coupled PDEs describing the dynamics of plant biomass, water concentration according to Klausmeier (1999), and one ODE describing the dynamics of toxic compounds (Cartení et al. 2012), with respect to the precipitation rate in the one-dimensional finite domain. Toxicity plays a key role in shaping plant communities even in the absence of water scarcity, particularly through autotoxicity (see e.g. Cartení et al. (2012); Consolo et al. (2024); Marasco et al. (2020, 2014); Mazzoleni et al. (2010, 2015), which can be attributed to soilborne pathogens (Bagchi et al. 2014) or autotoxic compounds (such as self-DNA) released during litter decomposition (Mazzoleni et al. 2010, 2015) explicitly including negative plant-soil feedback induced by toxicity via an additional differential equation leads to complex, rich dynamics not observed in classical models based on water-biomass interactions only, see e.g. Carter et al. (2024); Iuorio and Veerman (2021). First, we present analytical results on the location of Turing bifurcations, also considering the size of the domain, by performing a linear stability analysis that accounts for spatial-temporal perturbations of the homogeneous equilibrium state. Furthermore, we perform a numerical bifurcation analysis to track branches of both stable and unstable far-from-homogeneous equilibrium patterns, thereby discovering novel asymmetric patterns that arise due to secondary bifurcations of the initial Turing instability. This is the first time that such an analysis for such a vegetation model is provided, thus revealing regions of multi-stability and novel symmetric and far-from-the-homogeneous equilibrium asymmetric patterns.

## The mathematical model

The mathematical model analyzed in this paper was proposed by Marasco et al. (2014) to simulate the dynamics of three state variables, namely, the biomass *B*, the soil water *W*, and the toxic compounds *T*. Indeed, the positive (of water) and negative (of toxicity) feedbacks on plant biomass can explain the occurrence of different vegetation patterns also in non water-limited environmental conditions (Cartení et al. 2012; Klausmeier 1999).

The soil water *W* ($${\text {kg}}/{\text {m}}^2$$) increases uniformly due to the rain precipitation *p* and is reduced by the evaporation process at a rate *lW* and plants transpiration at a rate $$rB^2W$$. Moreover, the water diffuses in the soil with a diffusion coefficient $$D_W$$. The plant biomass *B* ($${\text {kg}}/{\text {m}}^2$$) grows at a nonlinear rate $$ rB^2W$$ according to water availability in the soil and dies due to a natural rate *d* and an extra loss induced by the presence of toxic compounds *T*. The intensity of toxicity depends on the plant sensibility, here parametrized by the parameter *s*. Plant lateral propagation is modelled by a dispersal term of a diffusion coefficient $$D_B$$. Toxic compounds *T* ($$\text {kg}/\text {m}^2$$) are produced by the dead biomass in a fraction *q* and decay by the decomposition process with a rate *k*, while they are washed out via precipitation with a rate *w*. The lateral movement of *T* is not considered, assuming that the toxic compounds do not move in the soil, i.e., the toxicity is removed from the system at a constant rate, without incorporating a diffusion process. These processes are formalized by the following system of two PDEs and one ODE.

1 $$\begin{aligned} \begin{aligned} B_t&=D_BB_{xx}+cB^2W-(d+sT)B \\ W_t&=D_WW_{xx} +p-rB^2W-lW \\ T_t&=q(d+sT)B-(k+wp)T , \end{aligned} \end{aligned}$$

with Neumann boundary conditions, i.e.,:

2 $$\begin{aligned} B_x(0,t)= B_x(L,t)=0, W_x(0,t)= W_x(L,t)=0, T_x(0,t)= T_x(L,t)=0. \end{aligned}$$

We consider a one-dimensional spatial domain, i.e. $x\in [0, L]$. In the model, the main bifurcation parameter is the precipitation rate, while the exact values of the other parameters are given in Table 1

**Table 1:** Values of model parameters (m-meter, d-day, kg-kilogram), see also Marasco et al. (2014)

parameter	Description	Values	Units
*c*	Growth rate of biomass *B*	0.002	${\text {m}}^4 {\text {d}}^{-1} {\text {kg}}^{-2}$
*d*	Death rate of biomass *B*	0.01	${\text {d}}^{-1}$
*k*	Decay rate of toxicity *T*	0.01	${\text {d}}^{-1}$
*l*	Water loss due to evaporation	0.01	${\text {d}}^{-1}$
*q*	Proportion of toxins in dead biomass	0.05	–
*r*	Rate of water uptake	0.35	$\text {m}^4 \text {d}^{-1} \text {kg}^{-2}$
*s*	Sensitivity of plants to toxicity *T*	0.2	$\text {m}^2 \text {d}^{-1} \text {kg}^{-1}$
*w*	Washing out of toxins by precipitation	0.001	$\text {m}^{2}\text {kg}^{-1}$
$D_B$	Diffusion coefficient for Biomass *B*	0.01	$\text {m}^2 \text {d}^{-1}$
$D_w$	Diffusion coefficient for water *W*	0.8	$\text {m}^2 \text {d}^{-1}$
*p*	Precipitation rate (bifurcation parameter)	[0, 2]	$\text {m}^{-2}\text {d}^{-1}\text {kg}$

## Linear Stability Analysis

In the following, we study the dynamics with respect to the precipitation rate parameter *p*. Initially, we seek for homogeneous solutions, setting the space and time derivatives in Eq. (1) equal to zeros, thus obtaining the following nonlinear algebraic system:

3 $$\begin{aligned} \begin{aligned} cB^2W-(d+sT)B&=0 \\ p-rB^2W-lW&=0\\ q(d+sT)B-(k+wp)T&=0 , \end{aligned} \end{aligned}$$

The above system (3) has a trivial bare soil solution $\textbf{u}_{h_1}=(B_{h_1},W_{h_1},T_{h_1})=(0,p/l,0)$. For a non-bare soil solution, i.e., when $B \ne 0$, we demonstrate the following proposition.

### Proposition 1

Let the nonlinear algebraic system (3). We define the functions $a_2(p)=sqcp+dr(k+wp)$, $a_1(p)=-(k+wp)cp$ and $a_0=(k+wp)dl$. Then, if the assumption

4 $$\begin{aligned} a_1^2-4a_0a_2>0 \end{aligned}$$

is satisfied, then the system (1) has two non-bare soil branches of solutions. Furthermore, these two branches bifurcate and disappear when

5 $$\begin{aligned} a_1^2-4a_0a_2=0. \end{aligned}$$

### Proof

We express the variables *W*, *T* as a function of *B* as

6 $$\begin{aligned} W=\frac{p}{(rB^2+l)}, \end{aligned}$$

7 $$\begin{aligned} T=\frac{1}{s} \left( \frac{cBp}{rB^2+l}-d \right) . \end{aligned}$$

Substituting the above in the third equation of the (7), we obtain a second order equation with respect to the biomass *B*, given by:

8 $$\begin{aligned} F(B,p)=a_2(p)B^2+a_1(p)B+a_0(p) =0 . \end{aligned}$$

For $\Delta = a_1^2-4a_0a_2>0$, Eq. (8) has two solutions with respect to the parameter *p*, defining two branches of a parabola given by

9 $$\begin{aligned} B_{h_{2,3}} =\frac{-a_1(p) \pm \sqrt{\Delta (p)}}{2a_2(p)}. \end{aligned}$$

The peak of the parabola results from $\Delta = a_1^2-4a_0a_2=0$. $\square$

### Remark 1

Substituting the values of the parameters from Table 1, the assumption Eq. (4) is satisfied iff $p>p_{c_0}=0.64$, while the second assumption given by Eq.(5) is satisfied when $p_{c_0}=0.64 \implies B=\frac{-a_1(0.64)}{2a_2(0.64)}=0.156$.

The homogeneous solutions with $B>0$ are defined from Eq. (9) and from Eqs (7), (6) are referred as $\textbf{u}_{h_2}=(B_{h_2},W_{h_2},T_{h_2})$ and $\textbf{u}_{h_3}=(B_{h_3},W_{h_3},T_{h_3})$, with $B_{h_2}>B_{h_3}$ respectively (see also Eq. (9)).

### Stability analysis of homogeneous solutions

In this section, we derive the stability conditions for homogeneous solutions, thereby studying the existence of Turing bifurcations that mark the onset of dynamical instabilities (Turing 1952, 1990). Our system given by Eq.(1) can be written in a compact form as:

10 $$\begin{aligned} \textbf{u}_t=R\mathbf {(u)}+{D}\textbf{u}_{xx}, \end{aligned}$$

where, $\textbf{u}=(B,W,T)$, ${R(\textbf{u})}=(f,g,h)$ with $f(\textbf{u})=f(B,W,T)=cB^2W-(d+sT)B$, $g(\textbf{u})=g(B,W,T)=p-rB^2W-lW$ and $h(\textbf{u})=h(B,W,T)= q(d+sT)B-(k+wp)T$. The matrix *D* is diagonal, with its main diagonal containing the diffusion coefficients, i.e.:

11 $$\begin{aligned} {D}=\begin{pmatrix} D_B & 0 & 0\\ 0 & D_W & 0\\ 0 & 0 & 0\\ \end{pmatrix}. \end{aligned}$$

(Note that $D_{3,3}=0$ since the third equation of the system (1) does not contain any diffusion term.) Thus, we study the stability of a given homogeneous steady state solution $\mathbf {u_{h_i}}$, $i=1,2,3$. When the propositions refer to a generic homogeneous steady-solution, we use the notation $\mathbf {u_0}=(B_0,W_0,T_0)$. When the propositions are applied to a specific steady-state solution, we use the notation $\mathbf {u_{h_i}}$. For the stability analysis, we introduce the perturbation $\mathbf {\delta u}= (\delta B,\delta W,\delta T)$ around the steady states, as $\textbf{u}= \textbf{u}_0+\mathbf {\delta u}$. Then, substituting the above into Eq.(10) and using first order Taylor expansion for the reaction term *R*(*u*), we obtain the following linearized equation of Eq.(10) around the steady state:

12 $$\begin{aligned} \mathbf {(\delta u)}_t={D} \mathbf {(\delta u)}_{xx}+J\mathbf {(u_0)}\mathbf {\delta u}, \end{aligned}$$

where $\mathbf {J(u_0)}$ is the Jacobian matrix:

13 $$\begin{aligned} {J(u_0)}= \left. \begin{pmatrix} f_B & f_W & f_T\\ g_B & g_W & g_T\\ h_B & h_W & h_T\\ \end{pmatrix} \right| _{u=u_0}. \end{aligned}$$

The perturbation $\mathbf {\delta u}$ should satisfy the Neumann boundary condition (2), implying

14 $$\begin{aligned} \mathbf {\delta u}={\textbf{C} e^{\lambda t}\cos \frac{n\pi x}{L}}, \end{aligned}$$

where $\textbf{C}=[c_1,c_2,c_3]^T$ is a constant vector. Then, the second-order spatial derivatives (the Laplacian) read

15 $$\begin{aligned} \mathbf {(\delta u)}_{xx}=-\left( \frac{n\pi }{L}\right) ^2 \mathbf {\delta u}, \end{aligned}$$

and the time derivative satisfies

16 $$\begin{aligned} \mathbf {(\delta u)}_{t}=\lambda \mathbf {\delta u}. \end{aligned}$$

Substituting the derivatives in Eq.(12), we obtain:

17 $$\begin{aligned} \lambda \mathbf {\delta u}= - {D} \left( \frac{n\pi }{L}\right) ^2 \mathbf {\delta u},+J\mathbf {(u_0)}\mathbf {\delta u}, \end{aligned}$$

or

18 $$\begin{aligned} \left[ - {D} \left( \frac{n\pi }{L}\right) ^2+J\mathbf {(u_0)}- \lambda {I}_{3}\right] \mathbf {\delta u}=\textbf{0}. \end{aligned}$$

Eq. (18) defines an eigenvalue-eigenfunction problem for the matrix ${A}=- {D} \left( \frac{n\pi }{L}\right) ^2+J\mathbf {(u_0)}$, and for a nontrivial solution (i.e. $\textbf{C} \ne \textbf{0}$), the following condition must be satisfied

19 $$\begin{aligned} \det [{A}-\lambda {I_3}]=\det \left[ - {D} \left( \frac{n\pi }{L}\right) ^2+J\mathbf {(u_0)}- \lambda {I}_3\right] =\textbf{0}. \end{aligned}$$

In our case, the matrix $\textbf{A}$ reads:

20 $$\begin{aligned} {A}= \left. \begin{pmatrix} f_B-D_B(\frac{n \pi }{L})^2& f_W & f_T\\ g_B & g_W-D_W(\frac{n \pi }{L})^2& g_T\\ h_B & h_W & h_T\\ \end{pmatrix} \right| _{u=u_0}, \end{aligned}$$

and at the steady state ${u=u_0}$, we get:

21 $$\begin{aligned} {A}= \begin{pmatrix} 2cB_0W_0-(d+sT_0)-D_B(\frac{n \pi }{L})^2& cB_0^2 & -sB_0\\ -2rB_0W_0 & -rB_0^2-l-D_W(\frac{n \pi }{L})^2& 0\\ q(d+sT_0) & 0 & qsB_0-k-wp\\ \end{pmatrix}. \end{aligned}$$

Eq. (19) defines the characteristic equation of matrix $\mathbb {A}$ of third order:

22 $$\begin{aligned} \begin{aligned} P(\lambda )&= \lambda ^3+c_2 \lambda ^2+c_1 \lambda +c_0\\&= \lambda ^3-{{\,\textrm{Tr}\,}}(A)\lambda ^2-\frac{1}{2}({{\,\textrm{Tr}\,}}(A^2)-{{\,\textrm{Tr}\,}}^2(A))\lambda -\det (A)=0, \end{aligned} \end{aligned}$$

i.e., $c_2=-{{\,\textrm{Tr}\,}}(A)$, $c_1=-\frac{1}{2}({{\,\textrm{Tr}\,}}(A^2)-{{\,\textrm{Tr}\,}}^2(A))$ and $c_1=-\det (A)$. We state now a general criterion for the stability of the homogeneous solution.

#### Proposition 2

(Stability criterion) The homogeneous steady state solution $\mathbf {u_0}=(B_0,W_0,T_0)$ of the reaction diffusion problem of Eq. (1), (2) is stable if the following conditions hold:

23 $$\begin{aligned} c_{2}>0,\quad c_{0}>0, \quad c_{2}c_{1}>c_{0}. \end{aligned}$$

#### Proof

If for each $$n\in \mathbb {N}$$, the roots of Eq. (22) lie on the negative complex semi-plane, then the homogeneous solution is stable. Otherwise, if one exponent passes the imaginary axis, i.e., if $$\Re (\lambda )>0$$, the homogeneous solution loses stability and becomes unstable. Using the Routh–Hurwitz stability criterion (Siettos and Bafas 2015; Sontag 1998) the homogeneous solution is stable if and only if $$c_{2}>0, c_{0}>0$$ and $$ c_{2}c_{1}>c_{0}$$. These conditions with the help of Eq. (22) can be written as:

24 $$\begin{aligned} {{\,\textrm{Tr}\,}}(A)<0, \det (A)<0, -\frac{1}{2}({{\,\textrm{Tr}\,}}(A^2)-{{\,\textrm{Tr}\,}}^2(A))\cdot {{\,\textrm{Tr}\,}}(A)>\det (A). \end{aligned}$$

$$\square $$

In the case of the trivial bare soil solution, i.e., for $(B_{h_1},W_{h_1},T_{h_1})=(0,p/l,0)$, the proof of stability is trivial.

#### Proposition 3

The bare soil steady state solution $\textbf{u}_{h_1}=(B_{h_1},W_{h_1},T_{h_1})=(0,p/l,0)$ of the reaction diffusion problem (1), (2) is always stable.

#### Proof

In this case, the matrix given in (21) takes the simple form:

25 $$\begin{aligned} {A}= \begin{pmatrix} -d-D_B(\frac{n \pi }{L})^2& 0 & 0\\ 0 & -l-D_W(\frac{n \pi }{L})^2& 0\\ qd & 0 & -k-wp\\ \end{pmatrix}. \end{aligned}$$

Thus, the eigenvalues of *A* are $\lambda _{1,n}=-d-D_B(\frac{n \pi }{L})^2<0$, $\lambda _{2,n} -l-D_W(\frac{n \pi }{L})^2<0$ and $\lambda _{3,n}=-k-wp<0$ for each $n\in \mathbb {N}$ ($k, w, d, p >0$). Hence, the bare soil solution is always stable. $\square$

### Existence of Turing instability

Based on proposition 3, the bare soil homogeneous solution $$\textbf{u}_{h_1}$$ is always stable and therefore cannot give rise to a Turing instability. On the other hand, the homogeneous solution $$\textbf{u}_{h_3}$$ =($$B_{h_3}, W_{h_3}, T_{h_3})$$ does not respect the necessary conditions for Turing instability, which imposes stability in the absence of diffusion. Hence, the only homogeneous solution which can give rise to Turing instability is $$\textbf{u}_{h_2}$$, thus in this section we investigate how the homogeneous solution $$\textbf{u}_{h_2}$$ (with $$B_{h_2}>B_{h_3})$$ in Eq. (9), can lead to more complex patterns. There are many different scenarios where the homogeneous solution loses stability. Since the characteristic polynomial is of third order, we can have one or two or even three real eigenvalues passing the imaginary axis. Another scenario is when two complex eigenvalues pass the imaginary axes. We state the following proposition.

#### Proposition 4

The homogeneous steady state solution $\textbf{u}_{h_2}=\mathbf {u_0}=(B_0,W_0,T_0)$ of the reaction diffusion problem given by Eq. (1), (2) loses stability if

26 $$\begin{aligned} c_{0}=-\det (A)=0. \end{aligned}$$

#### Proof

The simplest case of stability loss is when one leading eigenvalue passes the imaginary axis and becomes positive. When $\lambda =0$, $P(0)=0$. Thus, directly from Eq. (22), we obtain $c_0=-\det (A)=0$. $\square$

We study the branch of positive biomass solutions, i.e., for $B=B_{h_2}>0$. The solution $(B_0,W_0,T_0), B_0=B_{h_2}$ is given from Eq. (6)-(9). We simplify the matrix *A* given in (21), using Eq. (3). Dividing with *B* the first equation in (3), we obtain $cB_0W_0=d+sT_0$. From the second equation in (3), we get $rB_{0}^{2}+l=\frac{p}{W_0}$. Then, the *A* is simplified to:

27 $$\begin{aligned} {A}= \begin{pmatrix} cB_0W_0-D_B(\frac{n \pi }{L})^2& cB_0^2 & -sB_0\\ -2rB_0W_0 & -\frac{p}{W_0}-D_W(\frac{n \pi }{L})^2& 0\\ qcW_0B_0 & 0 & qsB_0-k-wp\\ \end{pmatrix}. \end{aligned}$$

#### Remark 2

In the case of the reaction diffusion problem (1), (2), the assumption in the proposition 4 reads:

28 $$\begin{aligned} \begin{aligned} F(p,n,L)=\left( cB_0W_0-D_B (\frac{n \pi }{L})^2\right) \cdot \left( -\frac{p}{W_0}-D_W(\frac{n \pi }{L})^2\right) \cdot (qsB_0-k-wp) \\ -2rcB_0^3W_0(-qsB_0+k+wp) +sB_0 \cdot \left( -\frac{p}{W_0}-D_W ( \frac{n \pi }{L})^2\right) \cdot qcW_0B_0=0 . \end{aligned} \end{aligned}$$

**Fig. 1:**
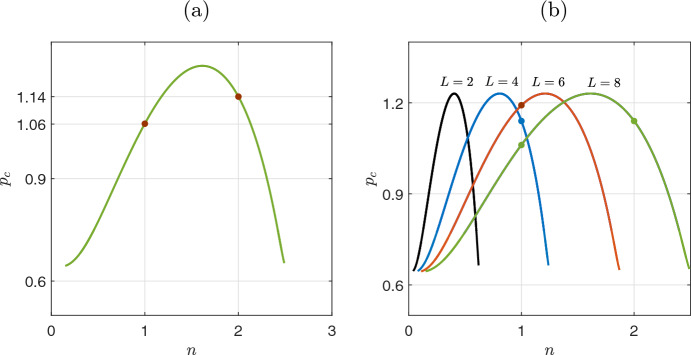
Critical values of the precipitation rate $p_c$ as a function of the natural number *n*, according to Eq. (28). **(a)** For $L=8$, the first critical value comes for $n=2$, which results for $p_{c_1}=1.14$. The second critical value results for $n=1$ and $p_{c_2}=1.06$ (both points marked with red filled circles). **(b)** The existence of critical values for the precipitation rate with respect to the domain size *L*. Here there are three scenarios: for higher values of *L* (e.g. close to 8), there are two critical values of the precipitation rate $p_c$ (equivalent Turing modes of instability) for $n=1$ and $n=2$. As *L* decreases, there is one critical value of $p_c$ (for $n=1$) and finally, when $L<L*=2.27$ there is no critical value of $p_c$ giving rise to Turing instability

For a constant *L*, Eq. (28) implicitly defines the parameter *p* as a function of the physical number *n*. Solving Eq. (28) in the case of $\mathbf {u_0}=\textbf{u}_{h_2}$ and for each value of $n, n=0,1,2,..$, we obtain the critical values of the parameter *p*. Fig. 1(a) shows the critical values $p_c=p_c(n)$ for $L=8$. For this size of the domain (specimen) only the modes for $n=1$ and $n=2$ result in the existence of a solution, while for $n=0$ and $n>2$ there are no critical values for $p_c$ ($p_c$ should also satisfy the conditions given by. (4), (5), i.e., $p_c>0.64$. The first critical value comes for $n=2$ and the first critical precipitation rate is $p_{c_1}=1.14$. The second one comes for $n=1$ and $p_{c_2}=1.06$ (marked with filled circles in Fig. 1(a)).

**Fig. 2:**
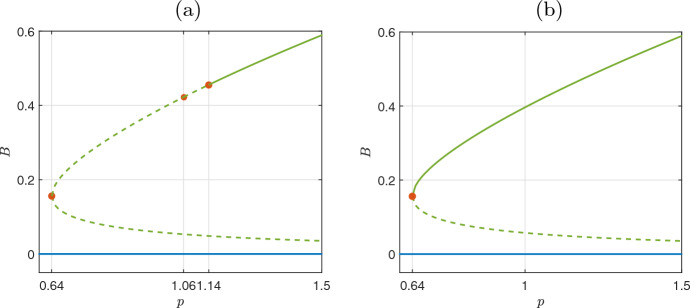
Bifurcation diagram of homogeneous solutions with respect to the precipitation rate *p*. Solid lines correspond to stable and dashed lines to unstable states, respectively. There are two sets of solutions. The first one is the bare soil branch ($B=0$) and the second set of homogeneous solutions with $B>0$ (as it is resulted from Eq. (9)). The second set consists of 2 branches which are bifurcated at the critical value $p_{c_0}=0.64$. Depending on the size of domain *L* the second set of solutions with $B>0$ shows different stability properties **(a)** For $L=8$, the upper branch loosing stability at (stable and unstable) On the stable branch of solutions with $B>0$ two critical values of *p* are marked with red circles $p_{c_2}=1.06$ and $p_{c_1}=1.14$. These values remark the onset of new inhomogeneous solutions, as we show in section 3.2**(b)** For $L=2$, the bifurcation curve has the same form as (a), however, since there is no Turing instability mode for $L =2<L^*=2.27$ the upper branch remains stable until the critical point of $p_{c_0}=0.64$ which bifurcates through a saddle node point

### Size Effect on the Turing Instability

The Turing eigenstability condition given by Eq. (28) allow us to investigate the size effect on the multiplicity of the homogeneous solution (with $B>0$). For different values of *L*, we repeat the previous procedure, for $n, n=0,1,2,..$, thus obtaining the corresponding critical values $p_{c_i}$. Fig. 1(b) shows the critical curves $p_c=p_{c,L}(n)$ for $L=2,4,6,8$. Higher values of *L* increase the width of the curve, as it is depicted in Fig. 1(b), introducing modes of instability (or equivalent, new types of inhomogeneous solutions). For, $L=8$, there are two critical modes for $n=1$ and $n=2$, while for $L=6$ and $L=4$ there is only one mode of instability at $n=1$. Finally, for $L=2$ there is no instability mode.

We can identify the critical size $L=L^*$ where the modes of Turing instability disappear. Demanding $F(p=0.64,n=1,L)=0$ we obtain the critical value $L^*=2.27$. For values $L<L^*$, there are no Turing instabilities, and the upper branch changes stability only at $p_{c_0}=0.64$ (see, proposition 1).

The impact of size *L* on the system dynamics can be represented in the bifurcation diagram of homogeneous solutions. Fig. 2 shows the bifurcations with respect to the precipitation parameter *p*, for two cases of the size *L*, one for $L=8>L^*$, Fig. 2(a), and one for $L<L^*$, Fig. 2(b). As we described in the case of $L=8$, the first critical parameter arises at $p_{c_1}=1.14$, and then the upper branch loses its stability. Then, this branch of solutions remains unstable. In the second case where $L<L^*$ the bifurcation curve is exactly the same, however there is a qualitative difference: since there is no Turing instability mode for $L<L^*$ the upper branch of Fig. 2(b) remains stable until the critical point of $p_{c_0}=0.64$ which bifurcates through a saddle node point.

Another information that we gain from the linear analysis is the shape of the solution near the criticality (i.e., near the values $p_{c_2}, p_{c_1}$). The shape also depends on the size *L*. If the first instability arises for $n=2$ (e.g. as in the case of $L=8$), then the solution near the critical value will be $\textbf{x}= \textbf{C}\cdot \cos (\frac{2\pi x}{L})$, with a spatial period $T=L$, which means that the profile is symmetric with respect to *L*/2. Instead, if the first instability appears at $n=1$ (which happens at low specimens *L*, e.g. for $L=6$ or $L=4$, see Fig. 1(b), then the solution (near the criticality) is $\textbf{x}= \textbf{C}\cdot \cos (\frac{\pi x}{L})$, with period $T=2L$. In this case, we have the half-period profile, meaning that the shape of the solution will be a skewed left or right half cosine.

Thus, we conclude with a general rule that if the first mode of the instability results from an even physical number $n_0$ (i.e., mod$(n_0,2)=0$), then the profile, near the criticality, is symmetric with respect to *L*/2, in the interval [0, *L*], while in the opposite case the profile is symmetric in the interval $[-L, L]$.

## Symmetry properties of the vegetation dynamics model

In this section, we discuss the symmetry problem for reaction-diffusion models, and, specifically, for the mathematical vegetation model (10). We also emphasise the connection between the symmetric properties of (10) with periodic boundary conditions (PBC), in the extended spatial domain $[-L, L]$ with the bifurcation properties of homogeneous steady-state solution with Neumann boundary conditions on [0, *L*] (NBC, i.e. Eq. (2)).

The reaction-diffusion model (10), with NBC has the property that for every non-homogeneous solution $\boldsymbol{u}(x, t)$ of Eq. (10), there exists a solution $\boldsymbol{u}(x',t)$, in which $x'$ is obtained from *x* by the action of a symmetry group G defined as:

29 $$\begin{aligned} x'=\gamma x, \quad \forall \gamma \in G. \end{aligned}$$

It can be easily shown that Eq.(1) are invariant under the $Z_2$ reflection symmetry:

30 $$\begin{aligned} x \rightarrow L-x. \end{aligned}$$

In the above system with NBC on [0, *L*], the solution can be extended in the domain $[-L, L]$, by reflection i.e.

31 $$\begin{aligned} u(-x)=u(x), \quad x\in [-L, L]. \end{aligned}$$

We comment here that solutions of Eq.(1) with NBC on [0, *L*] are in 1:1 with solutions satisfying the PBC on $[-L, L]$, having the symmetry:

32 $$\begin{aligned} u(-x)=u(x). \end{aligned}$$

To prove this, we consider a 2*L* periodic solution *u* with periodic boundary conditions (PBC). We differentiate the Eq. (32) to obtain $$ -u'(-x)=u'(x)$$, then for $$x=0$$ we have $$u'(0)=0$$ and for $$x = L$$ we get $$ -u'(-L)=u'(L)$$. Taking into account the 2*L* periodicity of *u*, we obtain also $$ u'(-L)=u'(L)$$, which implies $$u'(0)=u'(L)=0$$. The symmetry property of Eq. (32)can be described using group theory. Consider the two-element group $$B_N=\{1, R\}$$, where the reflection *R* is defined by

33 $$\begin{aligned} Rx=-x, \end{aligned}$$

then, functions which satisfy Eq. (32) are fixed points of $B_N$ (Crawford et al. 1989). We discuss in the next section the consequences of the periodic boundary condition (PBC) extension in the bifurcation.

### Steady states bifurcation using group symmetry

As we have already shown in the theoretical analysis of Section 3, the reaction-diffusion system admits at least one homogeneous steady-state solution. In addition to this solution, depending on the system’s parameter, the uniform solution may have bifurcations, see section 3. We state the following proposition, which characterises the bifurcation of the homogeneous solution (Crawford et al. 1989), p.65:

#### Proposition 5

Assume that the homogeneous steady state solution of the reaction-diffusion given by Eq. (32), with NBC undergoes a steady state bifurcation then, bifurcating solutions have a well-defined non-negative integer mode number $m>0$ and the bifurcation is a pitchfork.

#### Proof

To prove the proposition, firstly, we study the bifurcation problem on the interval $[-L, L]$ with PBC (which is equivalent: solutions that satisfying NBC are in 1:1 with solutions satisfying the PBC). The linearized equations of Eq. (10) about the homogeneous solution in the bifurcation point have no trivial kernel (e.g. Eq. (18)). It can be shown that the representation of O(2) on the kernel is $Z_m$. Therefore, bifurcation solutions are invariant under the translation

34 $$\begin{aligned} x \rightarrow x+2L/m. \end{aligned}$$

Returning on Neumann boundary conditions NBC, one has to restrict on the space ${\text {Fix}}(B_N)=\{z: Rz = z\}$ i.e, these solutions that are invariant under reflection $Rz=-z$. This symmetry can be expressed by the transformation

35 $$\begin{aligned} x \rightarrow x+2L/2m. \end{aligned}$$

We can apply the symmetry theory in the analytical results of section 3 and to guess the spatial patterns that appear in the bifurcation point of the homogeneous solution. For $L = 8$ we obtain the bifurcation mode for $m=2$ which near the bifurcation point, $p_{c_1}=1.14$, is $\textbf{u}_1=\textbf{u}_0+\delta \textbf{u}=\textbf{u}_0+\textbf{C}\cos (2\pi x/8)$. Using the aforementioned theory and the translation of Eq. (35), there is also the solution $\textbf{u}_2=\textbf{u}_0+\textbf{C}\cos (2\pi (x+2 \cdot 8/4)/8) = \textbf{u}_0+\textbf{C}\cos (2\pi x/8+\pi /2)=\textbf{u}_0-\delta \textbf{u}$, thus it is expected at the bifurcation point, the existence of 2 symmetric solutions with respect to the homogeneous $\textbf{u}_0$. Note that the second symmetric solution $\textbf{u}_2$ does not result form the reflection, $x \rightarrow L-x$ i.e. from Eq. (30). $\square$

## Numerical simulations and bifurcation analysis

In this section, we first investigate the dynamics of the system (1), (2) using numerical simulations. The previous analysis revealed the existence of critical values of the precipitation rate *p*, where the homogeneous solution loses stability due to Turing points. However, as discussed, the linear analysis does not provide any information for the type-profile of the new solutions (especially far from the bifurcation point). Furthermore, in many cases new types of inhomogeneous solutions arise from secondary bifurcations points far from the homogeneous solutions (see e.g. in pp.120 in Nicolis and Prigogine (1977)) leading to complex (ecological) patterns, which linear analysis can not predict. Thus, numerical simulations may be used as a first step to discover new types of solutions, and eventually multistability regions. However, as this way of analysis may discover the existence of only (some) stable solutions, in the next section, we complete the study by extracting all the branches of stable and unstable solutions by exploiting the arsenal of numerical bifurcation analysis.

### Numerical simulations

The reaction diffusion model (1), (2) is solved numerical using central finite differences in space, thus partitioning the domain [0, *L*] with $L=8$, into *N* equal intervals of size $h=\frac{L-0}{N}$ and $x_i=0+\frac{L-0}{N}\cdot i$, $i=0,1,2,...N$. For each time *t* we set $B(x_i,t)=B_i, W(x_i,t)=W_i, T(x_i,t)=T_i$. The model (1) is thus transformed to an ODEs system in time of $3 \cdot (N+1)$ equations, with state variables $B_i(t), W_i(t), T_i(t)$, which in compact form is:

36 $$\begin{aligned} \frac{d{\textbf{u}}}{dt}=\textbf{f}(\textbf{u},p), \end{aligned}$$

where the function $\textbf{f}=(f_{1,i},f_{2,i},f_{3,i})$, $i=1,2,..,N-1$ has the form

37 $$\begin{aligned} \begin{aligned} f_{1,i}&=c_B (B_{i-1}-2B_i+B_{i+1})+cB_i^2W_i-(d+sT_i)B_i \\ f_{2,i}&=c_W (W_{i-1}-2W_i+W_{i+1}) +p-rB_i^2W_i-lW_i \\ f_{3,i}&=q(d+sT_i)B_i-(k+wp)T_i , \end{aligned} \end{aligned}$$

where $c_B=\frac{D_B}{h^2}$ and $c_W=\frac{D_W}{h^2}$. Additionally, discretizing the Neumann boundary conditions with central differences, we decrease the number of ODEs equations to $3 \cdot N-3$. For example, we report the discretisation of the left boundary condition ($x=0$), which takes the form:

38 $$\begin{aligned} \begin{aligned} f_{1,0}&={c_B}\left( {2B_{1} - 2B_0} \right) + c{ {B_0}^2} W_0 - \left( {d + sT_0} \right) B_0\\ f_{2,0}&={c_W}\left( {2W_{1} - 2W_0} \right) + p - r{{ {B_0} }^2}W_0 - lW_0\\ f_{3,0}&=q\left( {d + sT_0} \right) B_0 - \left( {k + wp} \right) T_0 , \end{aligned} \end{aligned}$$

for $i=0$. Similarly, for the right boundary condition ($x=L$) and $i=N$, we obtain:

39 $$\begin{aligned} \begin{aligned} f_{1,N}&= {c_B}\left( {2B_{N-1} - 2B_{N}} \right) + c{{B_{N}}^2} W_{N} - \left( {d + sT_{N}} \right) B_{N}\\ f_{2,N}&={c_W}\left( {2W_{N-1} - 2W_{N}} \right) + p - r{{{B_{N}} }^2}W_{N} - lW_{N} \\ f_{3,N}&={q\left( {d + sT_{N}} \right) {B_{N}} - \left( {k + wp} \right) T_{N}}. \end{aligned} \end{aligned}$$

The resulting dynamical system of ODEs is solved using the Matlab ode23s solver suitable for stiff problems. For our computations, we have used $N=40$, and the default ode option for the relative and absolute error (relative error $10^{-6}$ and absolute error $10^{-6}$). Larger values of *N* resulted, for all practical purposes, quantitatively to same results.

**Fig. 3:**
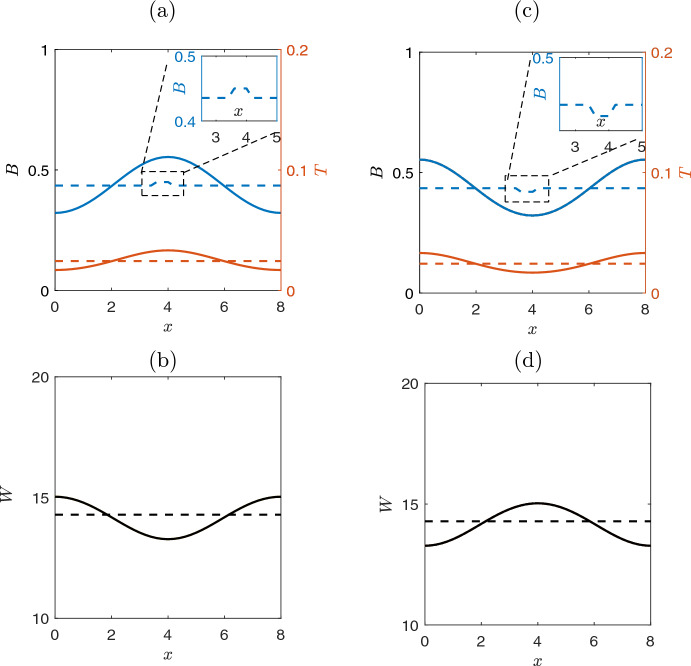
Evolution of the perturbed homogeneous solutions after the first bifurcation point of Fig. 2(a) at $p=1.1$. Dashed lines correspond to initial conditions, while solid lines depict the final steady state solution profile.**(a, b)** The biomass and toxicity profiles show a symmetric bell-shaped profile **(a)**, while the water shows an inverted bell-shaped profile **(b)**. The homogeneous solutions are also given for comparison purposes. The inset depicts the results obtained by perturbing upwards the homogeneous solution at the center of the domain (i.e., we initialise as: ${\textbf {x}}_{\text {init}}=1.1 \cdot {\textbf {x}}_{\text {hom}}$, $x\in [3.8, 4.2]$). The initial value and the steady state solution (dashed-dot) of the toxicity is depicted in the right y-axis. **(c, d)** The biomass and toxicity profiles corresponding to inverted bell-shaped profiles, when the perturbation of the initial conditions is oriented down. The inset shows the perturbation of the homogeneous solution oriented down, i.e., ${\textbf {x}}_{\text {init}}=0.9 \cdot {\textbf {x}}_{\text {hom}}$, $x\in [3.7, 4.2]$. **(d)** The water mass corresponding to a symmetric $\Lambda$ shape

For large values of the precipitation rate $p>p_{c_1}=1.14$, the ecosystem exhibits two stable homogeneous stationary states, one corresponding to the homogeneous vegetated state and the other corresponding to the bare soil solution. As the values of precipitation rate *p* decrease, and in perfect agreement with the linear analysis, the homogeneous vegetated solution loses its stability (through a Turing bifurcation at $p_{c_1}=1.14$, (see section 3).As a consequence, depending on the initial conditions, the system may converge to one of two new types of bell-shaped and inverted bell-shaped symmetric but inhomogeneous solutions for the biomass *B*. These two solutions are reported in Fig. 3(a,c), and they are obtained with initial conditions which are perturbations, in the center of the domain, in respect to the homogeneous solution: one positive (Fig. 3(a)) and one negative perturbation, see Fig. 3(c), respectively.

**Fig. 4:**
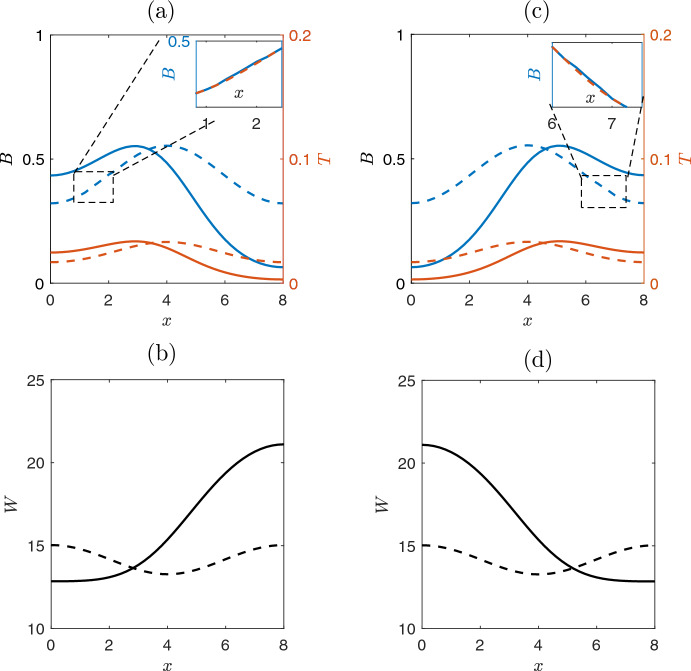
Profile of solutions close to a critical value where the system dynamics deviates from the bell-shaped solution of Fig. 3 i.e., simulations for $p=0.95$. Dashed lines correspond to the initial conditions, while solid lines depict the final steady states solutions. **(a)** Biomass, starting from initial condition close to solution of Fig. 3(a); after a transient period, the system converges to a skewed left inhomogeneous solution. The inset shows the initial condition, which is the bell-shaped of Fig. 3(a) (as red dash line) perturbed on the left side of the domain (i.e., ${\textbf {x}}_{\text {init}}=1.1 \cdot {\textbf {x}}_{\text {Bell}}$ for $x\in [1.1, 2.1]$).**(b)** Water mass dynamics exhibits a right asymmetric behavior. **(c)** A biomass skewed right inhomogeneous solution appears when the perturbation of the initial conditions is oriented right-up.**(d)** Water mass dynamics exhibits an opposite left asymmetric behavior

**Fig. 5:**
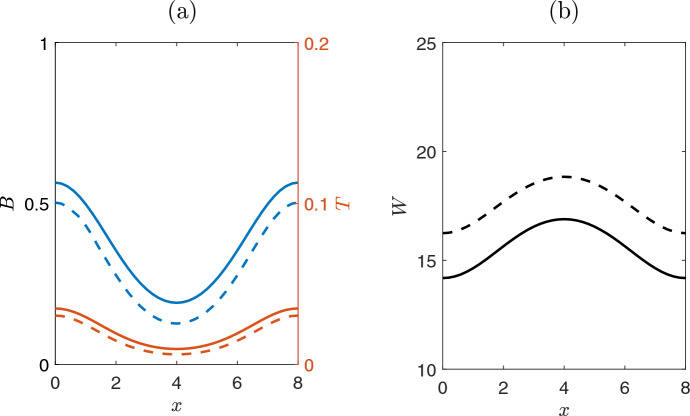
Persistence of the inverted bell-shaped solution. Temporal simulation for $p=0.95$. Dashed lines correspond to the initial conditions, while solid lines depict the final steady state solutions. In contrast to the bell-shaped solution (Fig. 4(a),(b) where the bell-shapes solution loses stability at $p=0.99$), the inverted bell-shaped solution keeps its stability until $p=0.91$, **(a)** for biomass and toxicity and **(b)** for water

A further decrease of the precipitation rate *p* value, results to another critical transition around $p_{c_{3}}=0.99$. In particular, the bell-shaped solution looses the stability and two asymmetric conjugate inhomogeneous solutions appear. These new couple of solutions are shown in Fig. 4 where the regime profiles are plotted for $p=0.95$, for different initial conditions. We consider as initial conditions, two perturbations of bell-shaped solution for biomass on the left or right (zoom box in Fig. 4(a) and Fig. 4(c), respectively). After a transient time, the system converges to two different regime stable solutions reported in Fig. 4(a),(c). We comment here that although the bell-shaped solution disappears after the critical point around $p_{c_{3}}=0.99$, the inverted bell-shaped solution remains stable until the value $p_{c_{4}}=0.91$ (see Fig. 5). For lower values of *p* (i.e., $p<p_{c_{4}}$) the inverted bell-shaped solution vanishes and the system exhibits only skewed left or skewed right solutions.

This kind of solutions exist for even smaller values of *p* and finally at some critical point (around $p=0.44$) the system depicts only the bare soil homogeneous solution ($B=0$ and $T=0$) which is permanent as $p \rightarrow 0$.

### Numerical bifurcation analysis

In order to systematically discover and trace both stable and unstable branches of steady state solutions, that are unreachable using the linear analysis of section 3 or with numerical temporal simulations resented in the previous section, and to accurately estimate the location of the critical points which mark the onset of phase transitions we resorted to the arsenal of numerical bifurcation theory. For the transformed system of Eq. (36) the steady states are computed as solution of equation:

40 $$\begin{aligned} \textbf{f}(\textbf{u},p)=0. \end{aligned}$$

The numerical bifurcation analysis is implemented with the aid of MatCont (Dhooge et al. 2003; Govaerts et al. 2022). The Matcont continuation algorithm is based on a predictor-corrector method (Dhooge et al. 2003; Govaerts et al. 2022). Suppose that we have detected a point $\textbf{x}_i=(\textbf{u}_{i},p_i)$ along the curve which is defined from eq. (40), also let $\textbf{v}_{i}$ a normalized tangent vector at $\textbf{x}_{i}$ , i.e. $\textbf{f}_x(\mathbf {x_i}) \cdot \textbf{v}_i=0$, and $||\textbf{v}_{i}||=1$. The computation of the point $\textbf{x}_{i+1}$ is made in two steps, first using a predictor (predicting a new point) and then, correcting the new point using Newton iterations.

As a predictor $\widetilde{\textbf{x}}_{i+1}$, we used a point on the tangent direction, i.e.:

41 $$\begin{aligned} \widetilde{\textbf{x}}_{i+1}=\textbf{x}_{i}+h \textbf{v}_{i}, \end{aligned}$$

where *h* is a small-selected step. The correction uses an augmented with one equation Newton scheme. We add the equation

42 $$\begin{aligned} g(\textbf{x})=(\textbf{x}-\widetilde{\textbf{x}}_{i+1})\cdot \textbf{v}_i=0, \end{aligned}$$

which is the well-known pseudo-arc-length continuation scheme, according to which, the final point results as the intersection of the hyperplane passing through $\widetilde{\textbf{x}}_{i+1})$ and the tangent predictor, i.e.,:

43 $$\begin{aligned} \textbf{x}^{k+1}= \textbf{x}^{k}-\textbf{F}_{x}^{-1}(\textbf{x}^k)\textbf{F}(\textbf{x}^k), \end{aligned}$$

with $\textbf{F}=(\textbf{f},g)^{T}$ and $\textbf{F}_x$ is the Jacobian matrix of $\textbf{F}$. The Newton-Raphson iterations termination criteria are the function and the step tolerance with tolerances set less than a specific value (here at $10^{-6}$) $||\textbf{F}(\textbf{x}^{k+1})|| < \epsilon _1$ and an additional accuracy condition $|| \delta \textbf{x} || < \epsilon _2$, where $\delta \textbf{x}$ is the last Newton-Raphson correction. The stability of the computed solutions is determined from the Jacobian matrix of eqs (37), (38), (39). Clearly, if all eigenvalues of the Jacobian matrix have negative real part, then the determined solution is asymptotically stable. On the contrary, if there is at least one eigenvalue with positive real part, then the computed solution is unstable.

**Fig. 6:**
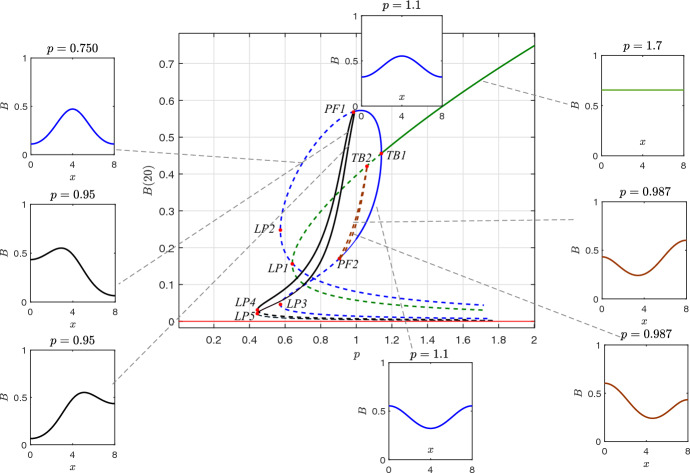
Bifurcations diagram with respect to the precipitation rate *p*. Solid lines correspond to stable states and dashed lines to unstable states. The system manifests rich nonlinear dynamics, with symmetric and asymmetric solutions, multistability, and symmetry-breaking bifurcations. The gray dashed lines depict the corresponding biomass spatial profiles of the steady state solutions. There are five branches: green and red which correspond to the homogeneous profiles, the blue branch which stands for the bell and inverted bell-shaped solutions, the black which is the skewed and left and right profile and the deep red unstable branch with asymmetric (almost inverted bell-shaped) solutions. The description and the exact positions of bifurcation points highlighted with labels are given in Table 2

### The bifurcation diagram

The bifurcation results are obtained from the discrete approximation eq.(36) of the original continuous model. Since the central difference scheme provides a second-order accurate discretization of spatial derivatives, the discrete bifurcation points converge to their continuous counterparts as the spatial step size decreases. In this limit, the discrete spatial modes approximate the continuous spectrum, and the qualitative bifurcation scenarios remain consistent. This becomes evident from the comparison of critical parameter values: both analytical and numerical schemes yield the same critical points (i.e., $p_{c_1}=1.14$ and $p_{c_2}=1.06$, see Fig.1,6).

Fig. 6 depicts the resulting bifurcation diagram. Characteristic profiles of the solutions along the branches are also shown as insets. Starting from $p=2$ and going downhill, the system shows only two branches of stable homogeneous solutions, one with $B>0$ and the second branch with $B=0$.

At a critical point $p_{c_1}=1.14$ (marked as *TB*1, in Fig. 6), corresponding to a Turing bifurcation, the homogeneous solution (with $B>0$) loses its stability and gives birth to two new inhomogeneous solutions of a bell-shaped and inverted bell-shaped (see insets and Fig. 3).

Decreasing the value of *p* further, the branch of homogeneous solutions remains unstable and on this branch, at the point $p_{c_2}=1.06$, a second point of Turing instability appears (marked as *TB*2, in Fig. 6).

**Table 2:** Critical values of bifurcation points (or tipping points) as they appear in Fig. 6

symbol in Figure 6	Description of symbols appears in fig.	critical value
*LP*1	Saddle node bifurcation of homogeneous solution	$p_{c_0}=0.64$
*TB*1	First Turing instability of homogeneous solution with $B>0$	$p_{c_1}=1.14$
*TB*2	Second Turing instability of homogeneous solution with $B>0$	$p_{c_2}=1.06$
*PF*1	Pitchfork bifurcation of the bell-shaped profile	$p_{c_3}=0.99$
*PF*2	Pitchfork bifurcation of the inverted bell-shaped profile	$p_{c_4}=0.91$
*LP*2	Bell-shaped saddle node bifurcation	$p_{c_5}=0.54$
*LP*3	Inverted bell-shaped saddle node bifurcation	$p_{c_5}=0.54$
*LP*4	Skewed left asymmetric profile saddle node bifurcation	$p_{c_6}=0.44$
*LP*5	Skewed right asymmetric profile saddle node bifurcation	$p_{c_6}=0.44$

At this second Turing bifurcation point (*TB*2), two new unstable branches of non-homogeneous solutions appear. Finally, the homogeneous unstable branch bifurcates through a saddle node bifurcation at $p_{c_0}=0.64$ (marked as *LP*1, in Fig. 6).

The bell-shaped and inverted bell-shaped solutions, which emerge from, *TB*1 are stable. The upper branch (with the bell-shaped patterns) remains stable until the critical point $p_{c_3}=0.99$ (marked as *PF*1 in Fig. 6). Then, the solution on this branch loses its stability and bifurcates with two new branches of inhomogeneous solutions, which are symmetrically conjugated. This type of secondary bifurcation can not be predicted from the linear analysis of the homogeneous solution. Remarkable, the inverted bell-shaped patterning keeps stability until $p_{c_4}=0.91$ (marked as *PF*2 in Fig. 6). Thus, the unstable branches emerging from *TB*2 connect *PF*2 and *TB*2 points. Finally, both unstable branches of bell-shaped and inverted bell-shaped patterns experience a saddle node bifurcation at the critical value $p_{c_5}=0.54$ (marked as *LP*2 and *LP*3 in Fig. 6).

Furthermore, at the point *PF*1, two new stable branches of inhomogeneous solutions arise. The profiles are skewed left and right solutions (see also Fig. 4). These branches lose stability under a saddle-node bifurcation, which takes place at the critical value $p_{c_6}=0.44$ marked as *LP*4, *LP*5 in Fig. 6. The profile of solutions is depicted with black color in the insets of Fig. 6.

Concluding, the system reveals a rich nonlinear dynamical behavior characterized by symmetry and symmetry-breaking bifurcations and the coexistence of multiple stable and unstable regimes. For $p \rightarrow 0$ the system exhibits only stable bare-soil solutions. Multistability is observed from *LP*4 to *PF*2 with three stable solutions (the bare-soil and two symmetrically conjugate solutions (depicted with black color lines in the insets of Fig. 6. Whereas from *PF*2 to *PF*1 the system provides four stable regimes (bare soil, two inhomogeneous symmetrically conjugate solutions and the inverted bell-shaped solution). From *PF*1 to *TB*1 there are three stable solutions (inverted bell-shaped, bell-shaped and bare soil solutions) and finally after *TB*1 we have the two homogeneous solutions, corresponding to the vegetated and soil solutions

**Fig. 7:**
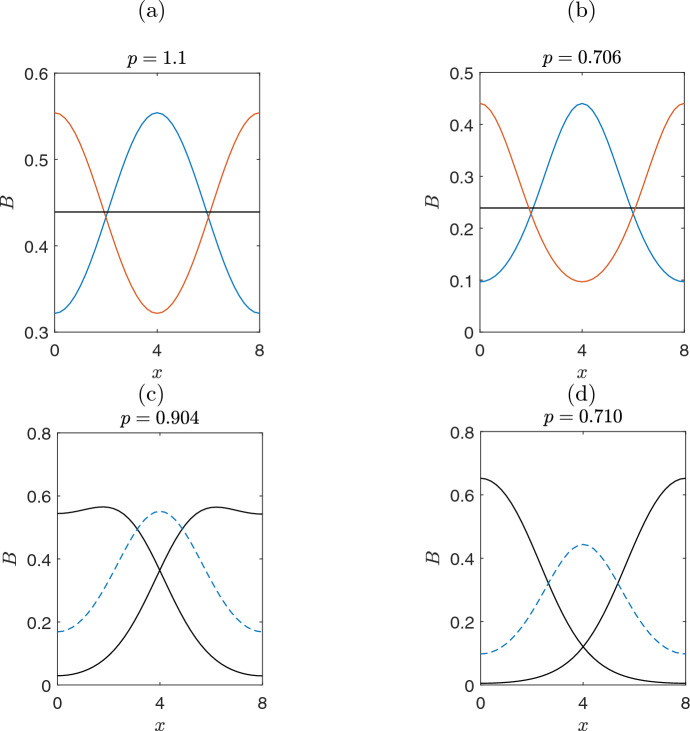
Symmetry and symmetry-breaking solutions. **(a)** Near the critical point, *TB*1 both bell-shaped and inverted bell-shaped solutions are symmetric with respect to the homogeneous solution (constant horizontal line with black colour). **(b)** Far from the *TB*1, the profiles are not any more symmetric. **(c,d)** Symmetry preserved along the branches of skewed-left and skewed-right inhomogeneous solution (black branches of Fig. 6). After the point, *PF*1, the two branches (black colour) preserve their symmetry

In Fig.7, we illustrate the symmetry breaking-symmetry of the solutions. As a consequence of the Turing bifurcation at the point $p_{c_1}=1.14$ (marked as TB1), there is a symmetry breaking of the homogeneous solution and two new solutions appear (bell-shaped and inverted bell-shaped profiles in Fig.7(a)). These solutions near the TB1 exhibit a symmetry, that one is the reflection of the other around the homogeneous solution. However, this symmetry is not preserved far from the TB1 (where the linearization is not valid and nonlinearity becomes significant), as it is shown in Fig.7(b). Furthermore,in Fig.7(c-d) are shown solutions arised from *PF*1 the symmetric between them, reflecting a conjugate symmetric pattern.

## Discussion

We performed bifurcation analysis of a biomass-water-toxicity model with respect to precipitation. The model consists of a set of two PDEs and one ODE that describe qualitatively the pattern formation in semi-arid zones as the precipitation decreases before the occurrence of desertification. We first performed a linear stability analysis for the solution branch of the homogeneous state to provide analytically: (a) the conditions for the appearance of Turing bifurcations that mark the onset of pattern formation, and, (b) the dependence of the Turing bifurcations on the size of the domain. Similarly to Mimura et al. (1991); Dey et al. (2022); Kabir and Gani (2022), we showed the dependence of solutions with respect to the size of the domain. Specifically, in Fig. 1, we have shown that as the size of the domain decreases the solution curve for the precipitation rate, i.e. Eq. (28) becomes narrow. This leads to a reduced number of bifurcation points of the homogeneous solution. Finally at a critical size of domain $L=L^*$ the Turing points disappear (see Fig. 1(b) the case of $L=2$). The aforementioned system behaviour is depicted in Fig. 2(a) for $L=8$, and Fig. 2(b) for $L=2<L^*$ where in (b) the homogeneous solution remain stable (no Turing patterns) until the saddle node bifurcation. In the case i.e. Fig. 2(a) at the critical points (Turing points), the system adds two inhomogeneous solution branches, which are symmetrical to the axis of the homogeneous solution. This is a known symmetry-breaking phenomenon, due to the Turing bifurcation, which, with the zero flux boundary conditions, has the characteristic of a pitchfork bifurcation (Dillon et al. 1994; Krause et al. 2021; Woolley 2022).

Here, we argue, based on numerical evidence, that the Turing-type symmetry breaking is fundamentally different from the symmetry-breaking bifurcations encountered in dynamical systems with $R^2$ symmetry. In particular, the numerical bifurcation analysis also reveals pitchfork bifurcations, which break the reflection symmetry induced by the boundary conditions. Different from the patterns arising from the Turing-Pitchfork-type bifurcation arising from zero-flux boundary conditions, here the reflection-conjugate patterns experience the same bifurcations and stability, and they always show up in pairs. As discussed also in Krause et al. (2021), while the linear stability analysis is formally valid around the Turing bifurcation from the homogeneous solution, it does not provide any information about possible subsequent bifurcations away from the uniform-equilibrium solution. In fact, we show that after the initial Turing symmetric instability, a secondary bifurcation arises which splits the solution branches into two distinct, unstable, asymmetric steady states, followed by a reverse asymmetric Turing bifurcation, in which the asymmetric equilibrium branches gain stability again. A similar mechanism has been observed in a two-layer model consisting of a pair of coupled reaction-diffusion equations (Yang and Epstein 2004). Regarding the formation of vegetation patterns, such asymmetric patterns have been observed in response to localized differences in soil-water availability (Tarnita et al. 2017).

As a limitation of our model, we refer to the toxic compounds: in our paper, toxicity is removed from the system at a constant rate, without incorporating a diffusion process. In a recent paper (Giannino et al. 2025) the authors proposed a cross-diffusion model for biomass and toxicity dynamics where water dynamics are neglected. Furthermore, a model has recently been developed that takes into account the following: a reaction-diffusion-advection model describing the dynamics of vegetation biomass and toxicity, which includes the effect of sloped terrains on the spatial distribution of these variables (Iuorio et al. 2024). Moreover, in the future, the movement of toxicity could be fully linked to the water diffusion.

We note that natural vegetation patterns are observed in two spatial dimensions, and translating 1D predictions into fully realistic 2D patterns is nontrivial. Nevertheless, one-dimensional PDEs serve as a useful simplification for uncovering the essential feedbacks (vegetation–water–soil) and for investigating through the bifurcation diagram criticalities and instability mechanisms that underlie pattern formation. Here, our analytical and numerical analysis is limited to the one-dimensional case, where studying the effects of the precipitation rate, domain size, and symmetry becomes more tractable. In the two-dimensional case with no-flux boundary conditions, the admissible wavelengths are given by $k^2=|k|^2=\pi ^2 ((n/L_1 )^2+(m/L_2 )^2)$ where $L_1$,$L_2$ denote the domain sizes in the x- and y-directions, respectively. As discussed in Murray (2003) (pp. 94–97), and in Marasco et al. (2014), such systems can exhibit a variety of spatial patterns, including spots, stripes, hexagonal structures, or even more complex patterns arising from secondary bifurcations. Moreover, conducting a numerical bifurcation analysis in two dimensions is computationally quite expensive or prohibitive due to the large size of the Jacobian matrix. Therefore, the 1D analysis is an essential complementary component of a multiscale modelling and analysis attempt: insights from 1D should be tested and extended with targeted 2D simulations and empirical comparison where feasible. For these reasons, the one-dimensional framework remains a widely adopted first step in the hierarchical modeling and analysis of vegetation pattern formation (see, e.g., Eigentler and Sherratt (2018); Curro et al. (2023))

Our analysis primarily focused on the role of precipitation; however, other parametersâ€”such as the decay rate of toxicity and the size of the domainâ€”are also ecologically relevant and merit further investigation. In this study, we derived the critical domain size for the onset of Turing instability. A comprehensive numerical bifurcation analysis with respect to domain size would require extensive parameter sweeps and thus remains an open question for future research.
